# Statistical Estimation of Accelerated Biological Brain Aging After Mild Traumatic Brain Injury in Older Adults

**DOI:** 10.1093/geroni/igaa057.3282

**Published:** 2020-12-16

**Authors:** Andrei Irimia, Jun Kim, Shania Wang, Hyung Jun Lee, Van Ngo, Sean Mahoney, David Robles

**Affiliations:** University of Southern California, Los Angeles, California, United States

## Abstract

Estimating biological brain age (BA) has the potential of identifying individuals at relatively high risk for accelerated neurodegeneration. This study compares the brain’s chronological age (CA) to its BA and reveals the BA rate of change after mild traumatic brain injury (mTBI) in an aging cohort. Using T1-weighted magnetic resonance imaging (MRI) volumes and cortical thickness, volume, surface area, and Gaussian curvature obtained using FreeSurfer software; we formulated a multivariate linear regression to determine the rate of BA increase associated with mTBI. 95 TBI patients (age in years (y): μ = 41 y, σ = 17 y; range = 18 to 83) were compared to 462 healthy controls (HCs) (age: μ = 69 y, σ = 18 y; range = 25 to 95) over a 6-month time period following mTBI. Across the initial ~6 months following injury, patients’ BAs increased by ~3.0 ± 1.2 years due to their mTBIs alone, i.e., above and beyond typical brain aging. The superior temporal and parahippocampal gyri, two structures involved in memory formation and retrieval, exhibited the fastest rates of TBI-related BA. In both hemispheres, the volume of the hippocampus decreased (left: μ=0.28%, σ=4.40%; right: μ=0.12%, σ=4.84%). These findings illustrate BA estimation techniques’ potential to identify TBI patients with accelerated neurodegeneration, whose rate is strongly associated with the risk for dementia and other aging-related neurological conditions.

